# Methane flux measurements along a floodplain soil moisture gradient in the Okavango Delta, Botswana

**DOI:** 10.1098/rsta.2020.0448

**Published:** 2021-11-15

**Authors:** M. J. Gondwe, C. Helfter, M. Murray-Hudson, P. E. Levy, E. Mosimanyana, A. Makati, K. B. Mfundisi, U. M. Skiba

**Affiliations:** ^1^ Okavango Research Institute, University of Botswana, P/Bag 285, Maun, Botswana; ^2^ UK Centre for Ecology and Hydrology, Atmospheric Chemistry and Effects, Bush Estate, Penicuik EH26 0QB, UK

**Keywords:** seasonal floodplains, methane emissions, methane oxidation, occasional floodplains, tropical wetland

## Abstract

Data-poor tropical wetlands constitute an important source of atmospheric CH_4_ in the world. We studied CH_4_ fluxes using closed chambers along a soil moisture gradient in a tropical seasonal swamp in the Okavango Delta, Botswana, the sixth largest tropical wetland in the world. The objective of the study was to assess net CH_4_ fluxes and controlling environmental factors in the Delta's seasonal floodplains. Net CH_4_ emissions from seasonal floodplains in the wetland were estimated at 0.072 ± 0.016 Tg a^−1^. Microbial CH_4_ oxidation of approximately 2.817 × 10^−3^ ± 0.307 × 10^−3^ Tg a^−1^ in adjacent dry soils of the occasional floodplains accounted for the sink of 4% of the total soil CH_4_ emissions from seasonal floodplains. The observed microbial CH_4_ sink in the Delta's dry soils is, therefore, comparable to the global average sink of 4–6%. Soil water content (SWC) and soil organic matter were the main environmental factors controlling CH_4_ fluxes in both the seasonal and occasional floodplains. The optimum SWC for soil CH_4_ emissions and oxidation in the Delta were estimated at 50% and 15%, respectively. Electrical conductivity and pH were poorly correlated (*r*^2^ ≤ 0.11, *p* < 0.05) with CH_4_ fluxes in the seasonal floodplain at Nxaraga.

This article is part of a discussion meeting issue 'Rising methane: is warming feeding warming? (part1)'.

## 1. Introduction

Global warming is associated with increasing atmospheric concentrations of greenhouse gases (GHGs) such as nitrous oxide (N_2_O), carbon dioxide (CO_2_) and methane (CH_4_) [1–3]. Methane is currently the second most abundant GHG in the atmosphere after CO_2_ [4]. In the period between 1800 and the 1990s atmospheric concentrations of CH_4_ increased; then stabilized at approximately 1775 ppb between 1999 and 2006 [5,6]. Renewed growth in global atmospheric CH_4_ concentrations started in 2007 [6–8]. The reasons for the stabilization and the renewed growth of approximately 6 Tg CH_4_ a^−1^ or approximately 3% increase in atmospheric CH_4_ concentration per year [9,10] since 2007 remain poorly understood due to seemingly contradictory findings, especially pertaining to the magnitudes of CH_4_ sources estimated using different methods, by various research works on the issue [6,9,10].

According to Dlugokencky and colleagues [8,10], the 2007 renewed growth in atmospheric CH_4_ concentration is consistent with abrupt increases in CH_4_ emissions from biomass burning and wetlands as well as a reduction in the tropospheric hydroxyl radical (OH) sink of CH_4_. Tropical and subtropical wetlands remain the world's largest natural source of CH_4_ to the atmosphere [11,12], accounting for 70% of the global wetland emissions budget [12,13]. However, estimates of natural CH_4_ sources and sinks, particularly in the South American, African and Asian tropics, remain poorly constrained, and with uncertain attribution to the various biogenic and anthropogenic sources [14,15], thereby hindering development and evaluation of regional and global CH_4_ budgets. Methanotrophic CH_4_ oxidation, the only known biological sink of atmospheric CH_4_, is also poorly understood [16] despite accounting for 4–6% of the total atmospheric CH_4_ sink [17].

**Figure 1.**
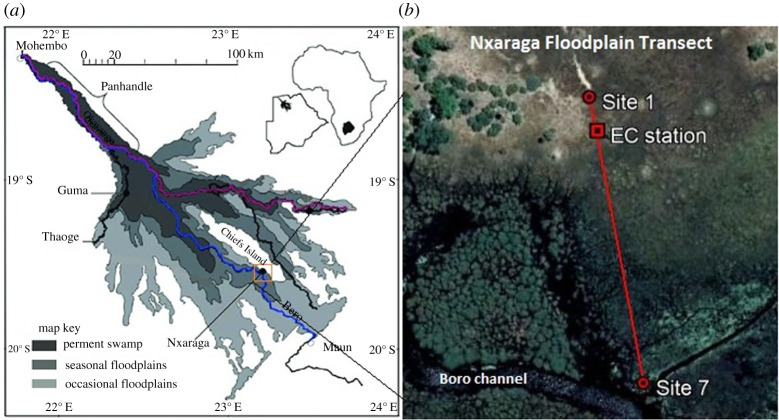
Map of the Okavango Delta (*a*) showing the study location and study transect across the seasonal floodplain at Nxaraga (*b*). The transect was oriented along the predominant southerly wind direction at Nxaraga. (Online version in colour.)

The aim of this study was to address the uncertainty of CH_4_ fluxes, particularly net CH_4_ fluxes and controlling environmental factors, from the alluvial Okavango Delta wetlands in Botswana, which is the world's sixth largest wetland. The Okavango Delta consists of permanent and seasonal wetlands bordering onto dry, occasionally flooded grassland and forest areas (figure 1*a*). We report the first chamber-based CH_4_ flux measurements along a soil moisture gradient in a seasonal floodplain in the Okavango Delta.

## 2. Material and methods

### (a) Study site

The Okavango Delta (figure 1*a*) is an alluvial wetland with a total area of approximately 22 000 km^2^ and a very low average gradient (1 : 3600) [18]. The Delta is recharged by flood-pulsed inflow of approximately 10 × 10^9^ m^3^ a^−1^ (range of 7–15 × 10^9^ m^3^ a^−1^; [19,20]) via the Okavango River which drains the central Angolan highlands located in a subtropical and humid climate with precipitation of up to 1300 mm a^−1^ [21,22]. An additional water input into the Delta comes from seasonal, erratic and localized convective rainfall over the wetland area averaging 490 mm a^−1^ (equivalent to 6 × 10^9^ m^3^ a^−1^) between December and April, peaking in February [20]. Due to the semi-arid conditions in the region, annual evapotranspiration in the Delta exceeds precipitation by a factor of more than 3, such that the system loses 98% of the total water inflow to the atmosphere. The remaining 2% exits the Delta as river outflow through the Boro/Thamalakane and Kunyere Rivers (figure 1) [19,23].

The Delta can be divided into three broad ecological zones based on their hydroperiods: the permanent swamp, the seasonal floodplains and the occasional floodplains (figure 1*a*). The permanent swamp, which covers a maximum area of about 3000 km^2^, consists of the Panhandle and the upper fan area, and is sustained by a base flow of approximately 150 m^3^ s^−1^ of the Okavango River [21]. The permanent swamp is dominated by dense vegetation stands composed of mainly *Cyperus papyrus* and *Phragmites australis* [20]. Most of the flood water in the swamp flows laterally through the dense vegetation [20], which, together with the low gradient, significantly decrease water velocity through the swamp, and consequently sediment and nutrient transport into the seasonal floodplains [24].

The flooding of the southern distal areas of the alluvial fan creates seasonal floodplains, which typically last for six to eight months depending on local summer rainfall and annual volume of inflow via the Okavango River. The seasonal floodplains can cover a total area of more than 3000 km^2^ [25], but flooding extents in excess of 6000 km^2^ have also been reported (see [26]). The flooding is followed by a burst of plant growth dominated by reeds (*Phragmites* spp.) and aquatic herbs (e.g. *Nymphaea* spp., *Potamogeton thunbergii*) in areas subject to longer and deeper floods (e.g. channels and lagoons), and sedges (e.g. *Cyperus articulatus*, *Schoenoplectus corymbosus*) in regularly inundated floodplain areas while grasses (e.g. *Miscanthus junceus*, *Panicum repens*, *Oryza longistaminata* and *Leersia hexandra*) dominate at the floodplain-woodland fringes [27–29]. Dry floodplain fringes are typically dominated by broad-leaved evergreen trees such as *Croton megalobotrys*, *Diospyros mespiliformis*, *Garcinia livingstonei* and *Ficus sycomorus* [30], while occasionally flooded areas (approx. once in a decade) in more distal locations are usually dominated by grasses such as *Urochloa* sp., *Eragrostis* spp. and *Aristida* spp. [27]. The plant species composition is, therefore, dependent on zone topography and hydroperiod across the floodplain areas [30,31]. The seasonal floodplains are heavily used for grazing and water by large numbers of wildlife species especially during the dry season when forage and water are scarce in the surrounding upland areas [32].

A substantial amount of water is lost to the atmosphere through evapotranspiration in the seasonal floodplains especially during maximum inundation. The evapotranspirative water loss by plants results in the accumulation of solutes (e.g. calcium, magnesium, potassium, silica and sodium) and nutrients (e.g. nitrogen and phosphorus) in soil water particularly under islands fringed by a variety of broad-leaved, evergreen trees and shrubs (see above). The evapotranspirative removal of solutes and nutrients from floodplain surface waters maintains the Okavango Delta as a freshwater system despite centuries of solute loading [33,34]. Most seasonal floodplains in the Okavango Delta experience regular fire activities during the dry winter period between May and September, which consumes most of the above ground dry vegetation and litter[35].

The high level of herbivory, low incident rainfall, infertile sandy soils and frequent fire events result in low accumulation of vegetation biomass in these seasonal floodplains compared to the permanent swamps [35]. In addition, the organic matter accumulated during wet periods may experience extensive aerobic decomposition during the dry season [36]. The arenosols consist predominantly of sands (up to 85% [37]) with an increase in peat and other organic material as distance to the river channel decreases thus creating an ‘O’ horizon in the lower seasonal floodplains. The predominant soils in this region are bright, well-drained sands or loamy sands (Haplic Arenosols) and dark greyish brown, poorly drained sandy loams or clays (Eutric Gleysols) [38].

**Figure 2.**
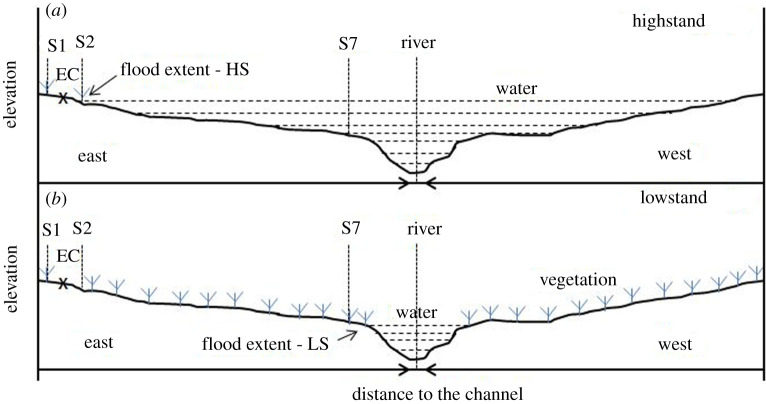
Schematic diagram of the highstand (*a*) and lowstand (*b*) flood extents in the seasonal floodplain at Nxaraga. The flood extent during a particular sampling campaign determined the length of that campaign's sample transect across the seasonal floodplain. The sample transect was longest at lowstand and shortest at highstand flood extents. For plant species and distribution across a seasonal floodplain see [27]. (Online version in colour.)

We established a spatial transect to study soil CH_4_ emissions using closed chambers in a seasonal floodplain at Nxaraga (figure 1*b*) on the southwest side of the Chief's Island in the Okavango Delta, Botswana. The floodplain is bounded to the west by the Boro River, which is one of the main channel systems and outlets of the Delta. The transect was constructed to span dry to waterlogged soils across the floodplain following the moving flood water edge (figure 2). Site 1 (19°32^′^52.40^″^ S, 23°10^′^44.75^″^ E) on rarely flooded dry soils was the only fixed site on the transect because it remained accessible throughout the sampling period (figure 1*b*). Site 2 slightly fluctuated but was basically located at the edge of the floodplain (where the water front reached) at highstand. Locations of Sites 3–7 depended on the length of the remainder of the transect which was equally portioned into additional two to five sites, partly determined by visual changes (especially wetness) in substrate conditions. The number of measurement sites, therefore, varied from a maximum of seven sampling sites at lowstand (during the peak of the dry season) to a minimum of four sampling sites at highstand (at maximum flooding extent) (figure 2). A total of 16 field campaigns were conducted over a 2-day period almost monthly from February 2018 to August 2020. Sampling was, however, not done regularly according to plan (monthly) due to unforeseen circumstances, primarily poor accessibility (by boat and vehicle) of the seasonal floodplain from Maun during certain seasons.

### (b) Measurements of CH_4_ fluxes

CH_4_ fluxes were measured using a closed dynamic chamber system comprising a transparent cylindrical polycarbonate chamber (388 mm dia., 305 mm high) placed on a pre-installed base, coupled to an ultra-portable GHG Analyzer (GGA, model 915-0011, Los Gatos Research (LGR), Mountain View, CA, USA). The performance of the generally robust GGA was checked at the UK-CEH for compliance with the World Meteorological Organization (WMO) requirements prior to the field campaign in the Okavango Delta, Botswana, using 3-point concentration standards calibrated relative to the WMO CH4-X2004 scale and WMO CO2-X2007 scale for CH_4_ and CO_2_, respectively. In the field, a set of three plastic chamber bases were installed at each site along the transect at least 12 h prior to gas measurements; these were removed soon after the measurements to avoid trampling by wildlife. The flow rate through the GGA absorption cell during measurements was 0.5 l min^−1^ and the chamber + tubing volume to surface area ratio was 30.5 cm^3^ cm^−2^. The air in the chamber was continuously mixed during measurements using a compact fan mounted on the lid of the chamber. The chamber operated as a closed system, meaning that the sample air was continuously withdrawn from the chamber headspace, passed through the GGA for simultaneous CO_2_, CH_4_ and H_2_O measurements and returned to the chamber. Each chamber measurement lasted 10 min and the GGA reported gas concentrations as dry mole fractions in ppm at 8 s intervals. The use of the GGA offers several advantages over laboratory gas chromatographs (GCs) commonly used to measure GHG fluxes. For instance, the sensitivity of infrared spectrometer technology used in the GGA is 500 times better than that of most GCs [39], accurate measurements of multiple gaseous components are taken in real time, the number of measurements obtained for calculating fluxes is many times larger, and a shorter enclosure time can be used. Diurnal variations were very small [40].

CH_4_ fluxes from the seasonal floodplain were also measured using eddy-covariance equipment deployed along the study transect (figure 1*b*) as a separate but complementary project reported elsewhere [41], except for comparison with the chamber-based fluxes.

### (c) Flux calculation

For each chamber measurement, the flux of CH_4_ was estimated from the time series of CH_4_ mixing ratio (nmol mol^−1^) with time. Because the response often deviates from linear, the initial rate of change d*C*/d*t* at *t *= 0 has to be inferred from the data using an appropriate model. Here, we fitted four models to the data (linear, quadratic, asymptotic and the HMR models [42]) and used the approach of Levy *et al*. [43] to choose the most appropriate estimate, based on goodness-of-fit criteria. Fluxes were calculated using the R statistical software (R Foundation for Statistical Computing, Vienna, Austria). For plotting and statistical analysis, Sigmaplot 14.0 software (Systat Software Inc., San Jose, CA, USA) was used.

### (d) Soil measurements

Soil temperature was measured next to the chamber base at a depth of approximately 10 cm using a Hygiplas digital thermometer (model GH628 with accuracy of ±1°C) during each chamber measurement event. After flux measurements were completed, soil samples (0–10 cm depths) were cored inside each chamber base, using a regular soil auger, and stored in airtight double-zipper plastic bags, yielding three sediment/soil cores at each site. These samples were analysed for soil pH, electrical conductivity (EC), soil water content (SWC) and soil organic matter (SOM) content using standard methods at the Environmental Laboratory, Okavango Research Institute, Maun, Botswana. Soil pH was determined potentiometrically according to Hendershot *et al*. [44] using a pH meter (WTW Inolab pH7110, Weilheim, Germany) after suspension in 1 : 2.5 (m/v) soil/water ratio. Soil EC was measured 2 h later on the same 1 : 2.5 (m/v) soil/water suspension using a WTW Inolab Cond7110 metre, Weilheim, Germany [45]. SWC and SOM were determined using gravimetric methods and reported on a dry weight basis. SWC, defined as the ratio of the mass of water present to the dry weight of the soil sample, was determined using homogeneously mixed wet soil subsamples weighed before and after drying to constant weight at 105°C for 24 h in a Scientific Series 2000 oven (RSA). SOM was estimated by weight loss-on-ignition [46]. A previously oven-dried (at 105°C) soil subsample (5.000 ± 0.005 g) was combusted in a Carbolite muffle furnace (UK) at 550°C for 2 h in pre-weighed clean ceramic porcelain crucibles, cooled to room temperature and reweighed. SOM (%) was calculated as the difference between the oven-dry soil mass and the soil mass after combustion at 550°C, divided by the oven-dry soil mass.

## 3. Results

**Figure 3.**
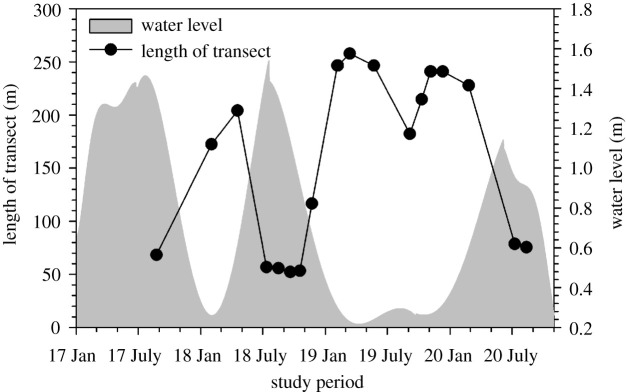
Variations in the length of the sampling transect due to seasonal flooding illustrated by water level in the bordering Boro channel at Nxaraga (figure 1*b*). Transects spanned Site 1 (at 0 m transect length) to the flood water front or edge of Boro channel (dark circles) as the water expanded or receded in the floodplain. Longest (e.g. greater than 200 m) and shortest (approx. 50 m) transects were, respectively, sampled during lowstand and highstand flood extents in the seasonal floodplain. See figure 2 for a schematic diagram of the floodplain cross-section at highstand and lowstand flood extents.

The length of the transect which was not inundated (figure 1*b*) ranged from approximately 52 m at maximum flooding (highstand) to 260 m at minimum flooding (lowstand) (figure 3). Soil temperature across the floodplain transect during the study period was recorded at 24.8 ± 0.8°C (mean ± s.e.).

**Figure 4.**
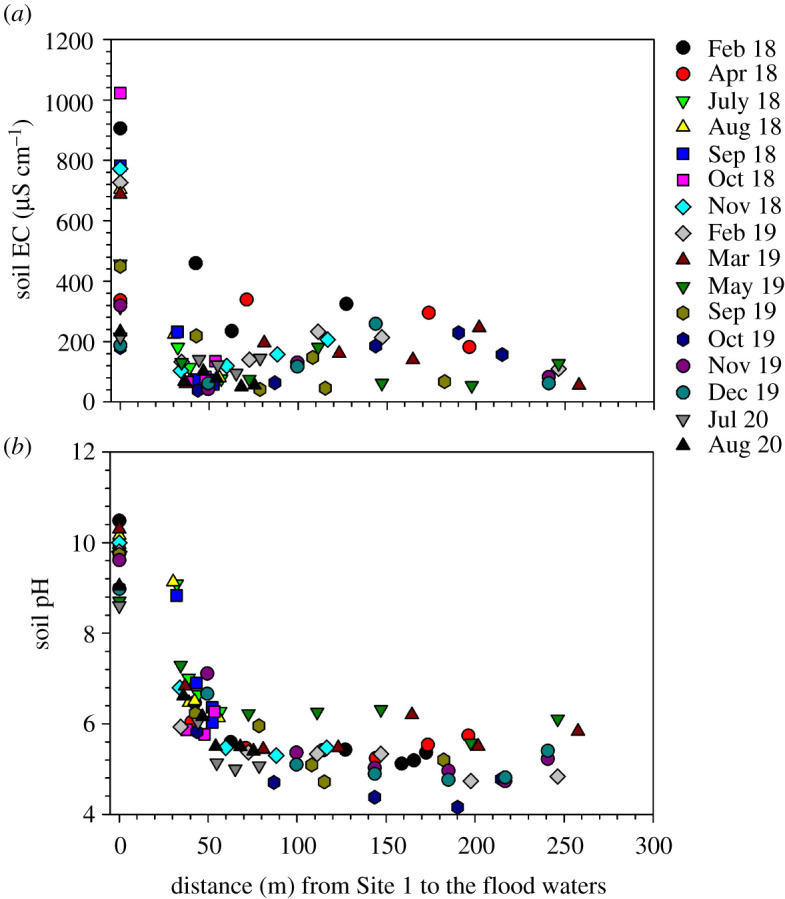
Variations of soil EC (*a*) and soil pH (*b*) along a transect (Sites 1–7) across a seasonal floodplain at Nxaraga. The data are the means of three replicates per sampling site for soil pH and soil EC obtained during the monthly 2-day sampling campaigns. The legend will be the same for the rest of the figures unless legend is provided for that figure. (Online version in colour.)

Site 1 was characterized by dry soils, and patches of salt deposits, common in central areas of most islands in the Okavango Delta, were conspicuous at the soil surface. Because of this, a much higher soil EC value (mean ± s.e.) of 578.3 ± 75.7 µS cm^−1^ was recorded at Site 1 compared to a mean of 91.6 ± 8.3 µS cm^−1^ observed at the other measurement sites along the floodplain transect. During 12 of the 16 monthly sampling campaigns, we observed a gradient of decreasing EC values along the transect from Site 1 to the flood water front (figure 4*a*). Soil pH showed an exponential decline from dry soils at Site 1 to the river channel (figure 4*b*). Soil pH was significantly higher (*p* < 0.05) at Site 1 (9.6 ± 0.1) than the mean pH of 5.7 ± 0.1 at the other sites along the floodplain transect.

**Figure 5.**
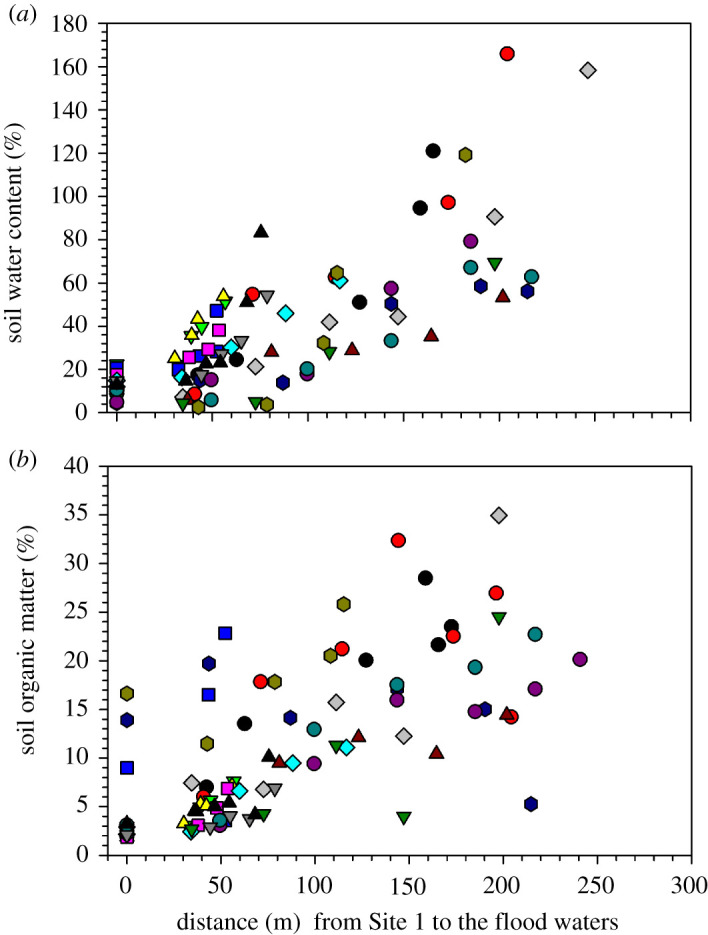
Variations of soil water content (*a*) and soil organic matter (*b*) along a transect (Sites 1–7) across a seasonal floodplain at Nxaraga. The data are the means of three replicates per sampling site for SWC and SOM obtained during the monthly 2-day sampling campaigns. See figure 2 for legend. (Online version in colour.)

**Figure 6.**
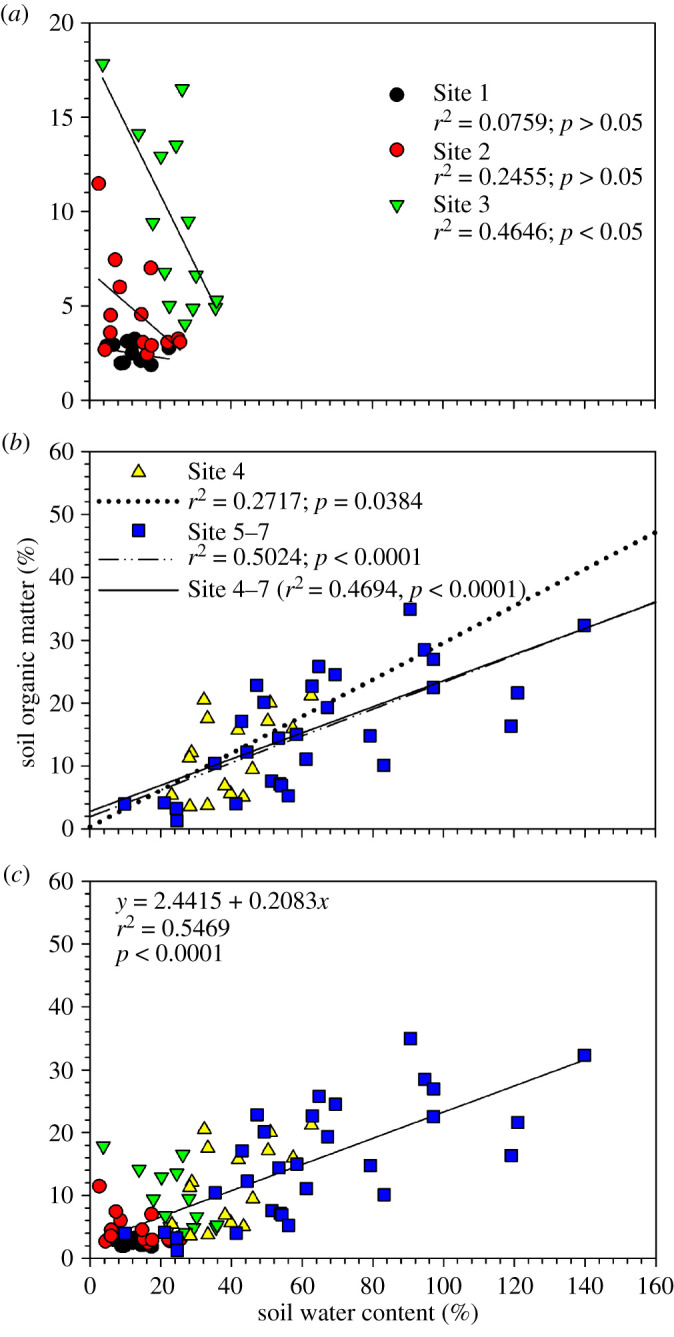
Relationship between soil water content (%) and soil organic matter (%) at Sites 1–3 (*a*), Sites 4–7 (*b*) and at all sites along the study transect (*c*) across a seasonal floodplain at Nxaraga. The data are the means of three replicates per sampling site for SWC and SOM obtained during the monthly 2-day sampling campaigns. The SWC–SOM relationship was assessed at site level in (*a*) and (*b*) because of the drought condition the seasonal floodplain experienced during the study period which might have affected the littoral sites more than the wetter sites near the Boro channel. (Online version in colour.)

SWC and SOM increased with distance along the floodplain transect to the river channel (figure 5*a*,*b*). The mean SWC and SOM recorded at Sites 1 and 2 were 13.40 ± 1.13% SWC and 4.90 ± 0.71% SOM, which increased to 73.28 ± 7.01% SWC and 16.12 ± 1.42% SOM between Sites 5 and 7. SWC and SOM values at littoral floodplain sites (Sites 1, 2 and 3 at 0, 40 and 70 m average distance along the transect, respectively) tended to be negatively correlated (*r*^2^ = 0.4646, *p* < 0.05 at Site 3; figure 6*a*), but positively correlated (*r*^2 ^= 0.4694, *p* < 0.05) at the lower floodplain Sites 4–7 (figure 6*b*). In general, aggregated SWC and SOM data from Sites 1–7 were positively correlated (*r*^2^ = 0.5469, *p* < 0.05; figure 6*c*).

**Figure 7.**
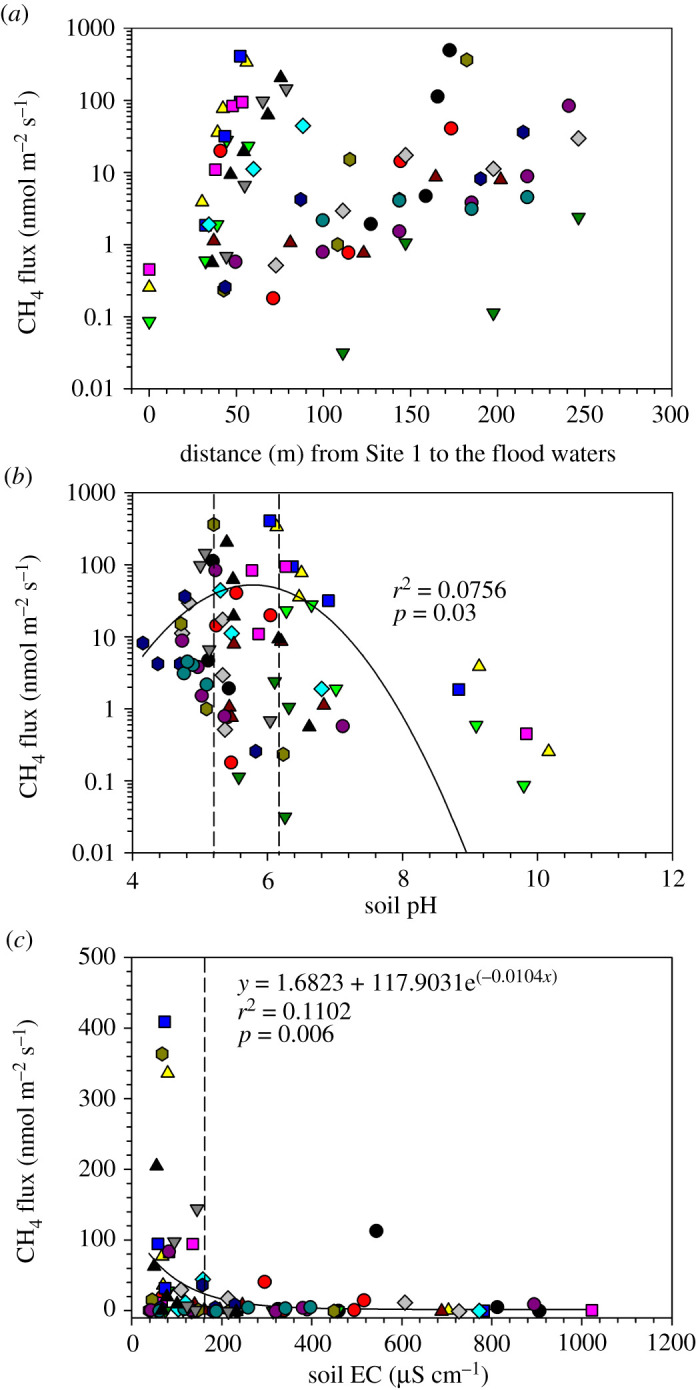
Variation of mean monthly CH_4_ fluxes with distance along the study transect (*a*) and its relationship with soil pH (*b*) and soil EC (*c*) in a seasonal floodplain at Nxaraga. The data are the means of three replicates per sampling site for soil pH, soil EC and soil CH_4_ fluxes obtained during the monthly 2-day sampling campaigns. The dashed lines in (*b*) and (*c*) indicate the optimum range of soil pH (pH 5.2–6.2) and soil EC (less than 160 µS cm^−1^) for soil CH_4_ flux in the seasonal floodplain at Nxaraga. See figure 2 for legend. (Online version in colour.)

Methane emissions increased from the littoral site (Site 1) to the flood water front during all sampling campaigns (figure 7*a*). The mean CH_4_ flux rate for all samples collected at Sites 2–7 during the whole sampling campaign between February 2018 and August 2020 was 44.89 ± 10.80 nmol m^−2^ s^−1^. By contrast, CH_4_ oxidation was primarily recorded at Site 1 (and sporadically at Site 2), and the mean flux was −0.79 ± 0.09 nmol m^−2^ s^−1^.

**Figure 8.**
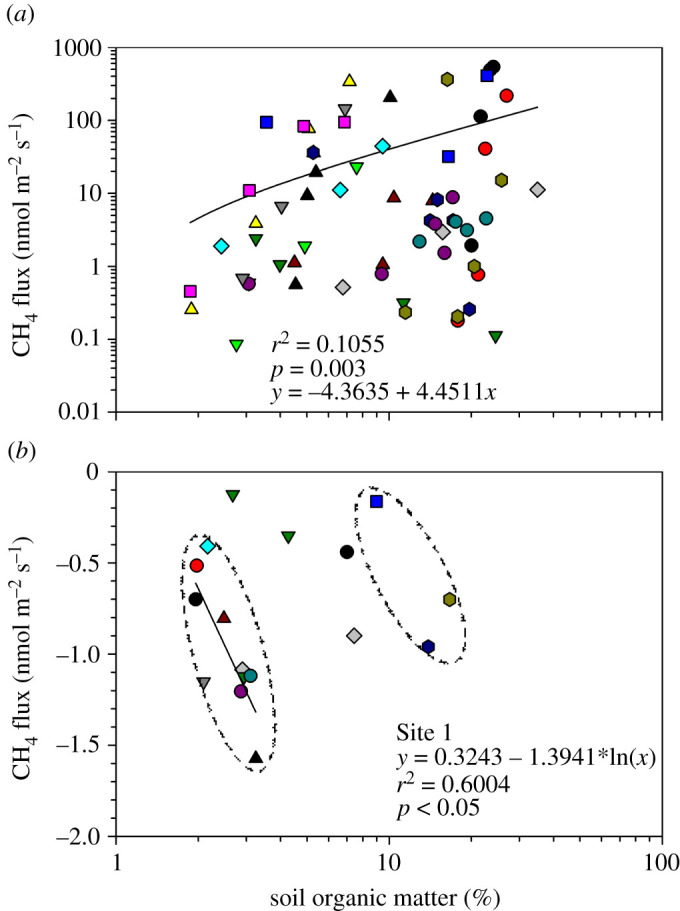
Relationship between soil organic matter (%) and CH_4_ emissions (*a*), and CH_4_ oxidation (*b*) in a seasonal floodplain at Nxaraga. The data are the means of three replicates per sampling site for SOM and soil CH_4_ fluxes obtained during the monthly 2-day sampling campaigns. The mean monthly CH_4_ oxidation fluxes in (*b*) were measured at Site 1 (in circles) and at Site 2 (outside the circles). See figure 2 for legend. (Online version in colour.)

**Figure 9.**
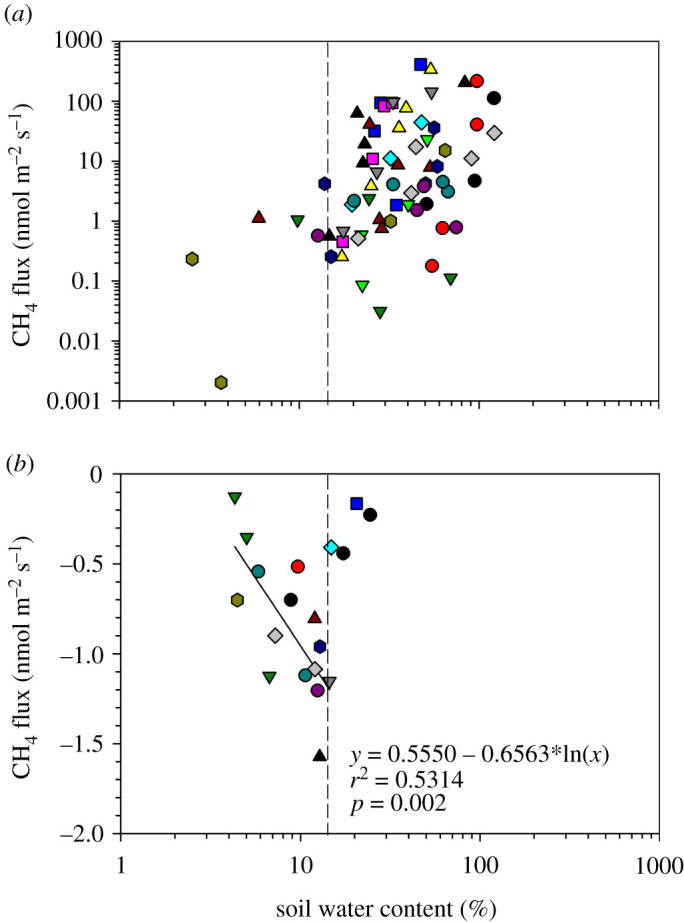
Relationship between soil water content and soil CH_4_ emissions (*a*) and CH_4_ oxidation (*b*) in a seasonal floodplain at Nxaraga. The data are the means of three replicates per sampling site for SWC and soil CH_4_ fluxes obtained during the monthly 2-day sampling campaigns. The dashed line indicates SWC at which soil CH_4_ oxidation (*b*) is optimum at 15% SWC. See figure 2 for legend. (Online version in colour.)

Soil pH and EC were poorly correlated (*r*^2^ ≤ 0.11) with CH_4_ fluxes (figure 7*b*,*c*). However, emissions were generally higher (greater than 100 nmol CH_4_ m^−2^ s^−1^) between pH 5.0 and pH 6.6 and EC of less than 200 µS cm^−1^. CH_4_ emissions showed a tendency to increase as SOM (figure 8*a*) and SWC (figure 9*a*) increased along the study transect. Similarly CH_4_ oxidation increased, especially at Site 1, as SOM and SWC increased from 2% to 5% (figure 8*b*) and 3% to 15% (figure 9*b*), respectively. Higher SOM and SWC favoured CH_4_ emission rather than oxidation.

**Figure 10.**
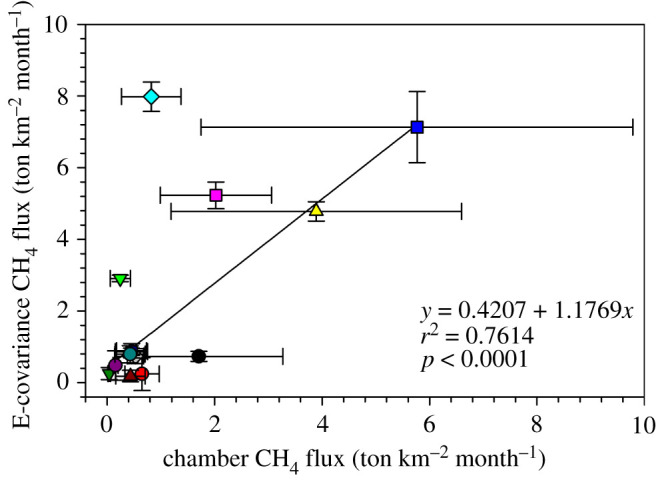
Correlation between chamber CH_4_ fluxes and EC-CH_4_ fluxes measured with closed dynamic chamber and eddy-covariance techniques, respectively, at Nxaraga seasonal floodplain in 2018 and 2019. The chamber flux data are the means of chamber Sites 2–7 obtained for each monthly day of sampling. The eddy-covariance flux is the conditional monthly mean of all available data points in the [130°, 270°] wind sector for which 90% of the measured flux originated within 200 m from the instrument mast. This condition was imposed to ensure that the flux footprint of the eddy-covariance system was limited to the portion of the floodplain where chamber sampling occurred. CH_4_ flux for November 18 (see legend in figure 4) was excluded for curve fitting. (Online version in colour.)

Soil CH_4_ fluxes measured by the closed chamber technique agreed reasonably well with their eddy-covariance counterparts in 2018 and 2019 (figure 10). To facilitate the comparison between the two techniques, eddy-covariance data were selected for monthly averaging if the flux footprint was consistent with the portion of the floodplain where chamber measurements took place. Without that condition on the extent of the flux footprint, the eddy-covariance fluxes were an order of magnitude larger than the chambers values in 2019, but still comparable in 2018.

## 4. Discussion

The data reported here spans the period from February 2018 to August 2020. During this period, the Okavango Delta experienced a significant reduction in annual water inflow via the Okavango River. For instance, the maximum inundation extent in the Delta estimated from MODIS imagery [47] declined from greater than 11 000 km^2^ in 2010–2012 to less than 3500 km^2^ in 2019 (http://www.okavangodata.ub.bw/ori/monitoring/flood_maps/). Consequently most seasonal floodplains, including Nxaraga floodplain, received little or no flooding especially during the 2019 flood season. The drought is likely to have affected biogeochemical processes in the floodplain soils including decomposition of organic matter, CH_4_ production and oxidation, and net fluxes [48,49].

The influence of pH on methanogenic and methanotrophic microbial activities is dynamic. For example, Valentine *et al*. [50] found a correlation between pH and potential CH_4_ production in laboratory experiments, while Moore & Knowles [51] found no relationship. At the global scale, Wen *et al*. [52] reported that temperature and soil pH are major controllers of methanogenesis. At Nxaraga seasonal floodplain, pH across the transect varied widely and ranged from pH 4.2 to 10.5. The pH decreased from dry soils at Site 1 to the edge of the Boro channel, as previously reported by Bonyongo *et al*. [53]. The variability was much larger across the lower floodplain area (pH 4.2–9.1 between Sites 2 and 7) than at the dry soil Site 1 (pH 8.6–10.5). According to Segers [54], the optimum pH for most methanogenic bacteria is 7.0. However, only 8% of the 112 soil pH measurements along the transect at Nxaraga were between pH 6.7 and 7.3. Approximately 73% of the pH measurements were lower than 6.7, which could mean that methanogens in the floodplain soils have adapted to the slightly acidic conditions such that the highest CH_4_ emissions were recorded at soil pH between 5.0 and 6.6 (figure 7*a*) [55]. Much higher soil pH values (8.6–10.5) at Site 1 were probably influenced by the salt deposits at the site (see EC below) and was the only site where CH_4_ was oxidized almost consistently throughout the study period, with an average flux of −0.79 ± 0.09 nmol m^−2^ s^−1^. Most methanotrophs operate around neutral pH, except for some species, which have adapted to high alkalinity situations, such as a highly alkaline soda lake (pH 9.5) in Central Asia [56]. Since salt deposits are common in forested island soils in the Okavango Delta, the methanotrophs are likely to have adapted to the high alkalinity in these soil environments.

Water EC, a measure of salinity, generally increases due to evapo-concentration as the flood water traverses the Okavango Delta to distal areas. A significant amount of water in floodplains around forested islands experiences a strong radial flow to the centre of islands induced by evapotranspiration by broad-leaved evergreen trees on the fringes of the islands. The process consequently concentrates solutes in the island fringe soils and in soil water underneath the islands. This permanent burial of solutes beneath islands is the main process that maintains the Okavango Delta as a freshwater ecosystem [33,34]. As the floodwaters recede, some of the remaining solutes are deposited on the rest of the floodplain soils causing an island-floodplain EC gradient (figure 4*a*). While salinity, defined as EC > 4000 µS cm^−1^, depresses both methanogenic and methanotrophic activities [57], the much lower EC values (this study) measured at Nxaraga floodplain sites did not seem to affect CH_4_ fluxes. Most of the higher fluxes (greater than 100 nmol CH_4_ m^−2^ s^−1^) were observed at soil EC < 200 µS cm^−1^, while high EC values, measured at the dry soil site, were accompanied by net microbial CH_4_ oxidation.

The importance of SWC and SOM in controlling CH_4_ emission and oxidation processes has been presented in literature [58]. CH_4_ emission is a net result of its production by methanogens and consumption by methanotrophs in anaerobic and aerobic soils, respectively [55,59]. Figures 8 and 9 show clear effects of SOM and SWC on both CH_4_ emission and oxidation in the seasonal floodplain at Nxaraga.

Although the range of soil CH_4_ fluxes along the study transect was large, the fluxes increased with SOM in the seasonal floodplain (figure 8*a*). A significant positive correlation (*r*^2^ = 0.1465, *p* < 0.05) was observed between natural-log transformed soil CH_4_ flux and SOM data (figure not shown). The high CH_4_ fluxes increased rapidly to a threshold of about 300–400 nmol m^−2^ s^−1^ at SOM contents of approximately 7–8% (figure 8*a*). Soil CH_4_ emissions and SOM correlate mainly because the SOM provides substrates for methanogenic CH_4_ production while its decomposition maintains anaerobic soil conditions by consuming dissolved O_2_ and other available alternative electron acceptors such as $\text{N}{{\text{O}}_{3}}^{-}$, Fe^3+^, Mn^4+^ and $\text{S}{{\text{O}}_{4}}^{2-}$ [60]. Soil CH_4_ emissions were found to be strongly correlated to SWC between the ranges 15–150% (figure 9*a*). An optimum SWC for CH_4_ emission has been, therefore, estimated at 50%, beyond which SWC appears to suppress soil CH_4_ emission by constraining the diffusivity of CH_4_ to the atmosphere (figure 9*a*). However, more sampling is needed to confirm the optimum SWC required for soil CH_4_ emissions by sampling multiple seasonal floodplains because it is an important environmental factor for modelling CH_4_ emissions in the Okavango Delta and potentially further afield.

Once emitted, the CH_4_ may remain in the atmosphere where it acts as a potent GHG with a global warming potential 28 times that of CO_2_, or it may diffuse into the dry soil matrix with subsequent oxidation to CO_2_ by methanotrophic bacteria. The widespread aerobic microbial CH_4_ oxidation which occurs primarily within the top 10 cm layer of undisturbed soils [61–63] is the only known biological sink of this GHG and accounts for 4–6% of the total global CH_4_ sink strength [17]. Soil CH_4_ oxidation, therefore, has a direct effect on net CH_4_ emissions from the environment. The rate of soil CH_4_ oxidation depends on the microbial activity of methanotrophs and the rate of diffusion of atmospheric CH_4_ within the soil profile [64,65]. The microbial CH_4_ oxidation process, just like CH_4_ production by methanogens, is regulated by a number of environmental factors such as SWC, SOM, temperature, pH and soil nitrogen content [59,66]. The rate of diffusion of atmospheric CH_4_ and O_2_ into the soil matrix for CH_4_ oxidation is controlled primarily by SWC and the physical soil structure (e.g. soil texture and compaction) such that waterlogged and fine-textured soils, for instance, have low gas diffusivity [59,65]. In fact, gas transport into the soil matrix has been suggested to be the main rate limiting factor for atmospheric CH_4_ oxidation in soils [67]. In the current study, although SWC and SOM at Site 1 varied within a narrow range, soil CH_4_ oxidation at the site increased with both SOM (figure 8*b*) and SWC (figure 9*b*). The optimum SWC for CH_4_ oxidation (where more negative fluxes were observed) in dry soils at Nxaraga was estimated at approximately 15% (figure 9*b*): lower and higher SWC values seem to supress CH_4_ oxidation, either by physiological water stress of methanotrophs at very low SWC or by restricting supply of both CH_4_ and O_2_ required for aerobic soil methanotrophic activity at higher SWC [58,65]. The optimum SWC of 15% for soil CH_4_ oxidation observed at Nxaraga is consistent with optimum SWCs for methanotrophic activities reported in previous studies: 11% SWC in Whalen *et al*. [68] and 20% SWC in Castro *et al*. [69]. The variation has been suggested to primarily depend on soil type [70]. Figure 9*a* further suggests that SWC values between 15% and 50% enhance CH_4_ emission, rather than oxidation, from the seasonal floodplain soils because as SWC increases, it simultaneously creates conducive anaerobic conditions for CH_4_ production when substrates are available and lowers its oxidation rate by restricting O_2_ supply into the soil [55,59]. It was not possible to estimate the optimum SOM for soil CH_4_ oxidation from the data currently available for the Delta, and therefore calls for more research on the effect of SOM and other environmental factors, including soil inorganic nitrogen concentration, not assessed in thisstudy.

## 5. Upscaling of CH_4_ emission and oxidation rates to the whole Okavango Delta

The mean CH_4_ emission rate measured by closed chambers at Nxaraga (44.89 ± 10.80 nmol m^−2^ s^−1^, equivalent to 2.61 ± 0.62 mg CH_4_ m^−2^ h^−1^) is comparable with emissions from several tropical wetlands across the world (range 0.003–40.4 mg CH_4_ m^−2^ h^−1^ and mean of 7.67 mg CH_4_ m^−2^ h^−1^), including the Congo River Basin with a CH_4_ flux of 4.41 mg m^−2^ h^−1^ [71]. The mean CH_4_ flux rate at Nxaraga is also comparable to fluxes from 71 northern, temperate and subtropical wetlands where mean fluxes were reported at 2.01 ± 0.16 mg CH_4_ m^−2^ h^−1^ for subtropical, 3.03 ± 0.05 mg CH_4_ m^−2^ h^−1^ for boreal, 4.54 ± 0.19 mg CH_4_ m^−2^ h^−1^ for temperate and 4.68 ± 0.26 mg CH_4_ m^−2^ h^−1^ for subarctic wetlands [72]. A more recent analysis of global natural wetlands estimated CH_4_ emissions at 2.01 mg CH_4_ m^−2^ h^−1^ (range 1.37–2.43) [15]. Similarly, the soil CH_4_ oxidation rates (0.046 ± 0.005 mg m^−2^ h^−1^ observed at Nxaraga are also comparable to mean CH_4_ oxidation rates of 0.13–2.02 mg m^−2^ h^−1^ recorded in northern, temperate and subtropical wetlands [72].

Although uncertainties of these flux estimates are large, the similarity in CH_4_ flux rates observed in different wetlands and parts of the world is noteworthy. Research on CH_4_ fluxes from wetlands is generally skewed towards arctic and temperate climate wetlands, with fewer studies from tropical and subtropical climate zones. This imbalance needs to be addressed in order to better model the global CH_4_ budget.

Chamber fluxes are representative of small-scale biophysical and biogeochemical processes and spatial heterogeneity can make upscaling to plot or landscape scales challenging, although strong agreements between upscaled chamber fluxes and fluxes derived by other techniques such as eddy-covariance have been reported [73–76]. In this study, a good agreement (*r*^2^ = 0.8102, *p* = 0.0002) was also observed using monthly averaged areal CH_4_ fluxes in 2018 and 2019 from the chamber and eddy-covariance techniques (figure 10).

Gumbricht *et al*. [25] estimated the total area of the Okavango wetland at 13 693 km^2^, about 22%, 24% and 54% of which are permanently, seasonally and occasionally flooded areas of the system. These figures have been recently revisited to take into account the climatic and developmental changes the Okavango basin, which includes the Delta, has experienced over the past two decades. While seasonal floodplains are inundated almost annually, occasional floodplains are flooded only during high floods, which occur approximately once in a decade, and can, therefore, be considered as dry areas capable of CH_4_ oxidation as observed at inland Site 1 in this study. Assuming that Site 1 at Nxaraga (where CH_4_ oxidation > CH_4_ production) is a proxy for the occasionally flooded wetlands and that Sites 2–7 (where CH_4_ production > CH_4_ oxidation) are representative of seasonal floodplains in the Okavango Delta, we estimate the net CH_4_ emission for these two hydrological zones to be of the order of 0.072 ± 0.016 Tg a^−1^ with net emissions at seasonal swamps of 0.075 ± 0.018 Tg a^−1^ and net oxidation in occasional swamps of the order of 2.817 × 10^−3^ ± 0.307 × 10^−3^ Tg a^−1^. These estimates suggest that the biological CH_4_ sink in the occasional floodplain soils in the Delta is small as it accounts for only 4% of the total CH_4_ emissions in the seasonal floodplains in the wetland. The fraction of the atmospheric CH_4_ associated with the biological sink in the Okavango Delta is, however, comparable to the global average sink of 4–6% [17]. There is, however, need for more research in soil CH_4_ oxidation in the Delta since the current study sampled only one site that may not adequately represent the vast areas of the occasionally flooded wetlands of the system.

## 6. Conclusion

This study estimated chamber-based CH_4_ emission and oxidation rates in a seasonal floodplain in the Okavango Delta at 44.89 ± 10.80 nmol m^−2^ s^−1^ and 0.79 ± 0.09 nmol m^−2^ s^−1^, respectively. Although measurements were done during a relatively dry period for the Delta, the observed CH_4_ fluxes are comparable to fluxes in other wetlands across the world. The observed CH_4_ oxidation which was upscaled to the whole dry occasionally flooded swamp area of the Okavango Delta accounted for approximately 4% of the total CH_4_ emissions from the Delta's seasonal floodplains. The Delta's CH_4_ sink due to methanotrophic activity is also comparable to the global average biological sink of 4–6%. As in other wetlands, SWC followed by SOM were found to be the main environmental factors controlling CH_4_ fluxes in seasonal swamps in the Delta. Maximum CH_4_ emission and oxidation rates in the seasonal floodplain at Nxaraga were observed at 50% and 15% SWC, respectively. EC and pH were not correlated with CH_4_ fluxes in the seasonal floodplains. Upscaling of CH_4_ oxidation fluxes were achieved from measurements at only one dry soil site. Future studies should, therefore, attempt to measure CH_4_ oxidation fluxes at several dry soil sites in different drylands (e.g. grasslands, woodlands, forests) of the Okavango Delta.

## Data accessibility

The data that are used in this paper are provided as electronic supplementary material.
